# Temporal meal patterns in the Swedish population and associations with socio-demographic variables and nutrient intakes: a cross-sectional study

**DOI:** 10.1017/S0007114525103462

**Published:** 2025-05-28

**Authors:** Jenny Schultz, Lotta Moraeus, Anna Karin Lindroos, Eva Warensjö Lemming

**Affiliations:** 1 Department of Food studies, Nutrition and Dietetics, Uppsala University, Uppsala, Sweden; 2 Division for Risk and Benefit Assessment, Swedish Food Agency, Uppsala, Sweden; 3 Medical Epidemiology, Department of Surgical Sciences, Uppsala University, Uppsala, Sweden

**Keywords:** Meal patterns, Chrononutrition, Meal frequency, Meal timing, Nutrient intake

## Abstract

Timing of food intake seems to impact metabolism and circadian rhythms, and eating in synchronisation with the rhythms has been suggested to be favourable for health. This study aims to evaluate temporal meal patterns in the Swedish population and explore differences between population groups. Further, to investigate whether temporal meal patterns are associated with energy and nutrient intake, data were sourced from two national dietary surveys Riksmaten Adolescents 2016–2017 and Riksmaten Adults 2010–2011, with a total of 4763 participants. Food intake and temporal meal patterns were measured with 3- to 4-day food diaries and/or 24-hour recalls. The average meal frequency was 4·2 eating occasions (sd 0·9) per day for adolescents with an eating window of 11·9 h (sd 1·7). For adults, it was 4·6 (sd 1·1) eating occasions and an eating window of 12·0 h (sd 1·9) Meal frequency was positively associated with energy intake in both adolescents (*r* = 0·47) and adults (*r* = 0·51). Meal frequency was higher with age, and adolescents skipped breakfast more often, and had a later energy distribution than adults. A higher eating frequency and eating breakfast are associated with a higher absolute intake of whole grains, as well as Vitamin D and folate. A higher eating frequency makes it more likely to reach nutrient requirements. However, a higher eating frequency was also associated with a higher intake of free sugars. The findings can serve as reference data for temporal meal patterns in the Swedish context and also show differences within a population, which can be valuable insights for public health nutrition.

Nutrition research has for the last decades focused mainly on what we eat and less on when we eat. Temporal meal patterns refer to the timing, regularity and frequency of food intake during the day. Their association with the circadian rhythms and metabolic health are studied in the emerging field – chrononutrition^(1)^. Temporal meal patterns (hereafter referred to as meal patterns) vary widely between cultures and countries and have evolved over time^(2–5)^.

Timing of food intake are hypothesised to be important for our health possibly due to shifts in metabolic responses of food intake at different times of the day^(1,6)^. The circadian rhythm is important for many physiological processes and plays a role in our eating, appetite regulation, digestion and metabolism. Our food intake is driven by, but also feeds back to, the circadian clock. Therefore, the timing of food intake might impact metabolism and the circadian rhythm in the body^(1,6)^. Research suggests that eating in synchronisation with the circadian rhythms is favourable for health^(1,7)^. Research on shift workers has shown a higher risk of developing the metabolic syndrome, possibly due to a misalignment with the circadian rhythms^(8–10)^. Further research has found associations between meal patterns, such as meal frequency, late energy intake (EI), regularity and breakfast skipping and CVD risk, BMI, diet quality and nutrient and EI, but research is inconsistent^(11–22)^.

Few countries have public health recommendations on meal patterns today, but Sweden used to recommend the population to eat breakfast, lunch, dinner and one or two snacks^(23)^. This recommendation was based on practical experience and cultural values rather than on research and scientific evidence. Reviews for the latest Nordic Nutrition Recommendations 2023 and the Dietary Guidelines for Americans 2025–2030 have also concluded that the evidence for providing specific recommendations on meal patterns is limited and inconclusive^(24–27)^.

Previous research has stated that there is a need for alignment in definitions and methodology in this research field, for example, for the definition of a meal, since study results have been shown to differ by definition^(28)^. A consensus on meal pattern definitions is important for comparisons and interpretation of results in the field. This study aims to align with definitions used in previous research^(20,29)^.

Meal patterns differ by country and culture, but might also vary by age, sex and demographic and social factors, which have not been much studied as of today. There is a lack of data on the meal patterns of the Swedish population on a national level, and further studies on larger populations are also needed to understand this and to forward the research field. This study aims to explore meal patterns; meal frequency, eating window, regularity, early or late energy distribution, largest meal of the day and breakfast skipping in two national, representative Swedish populations, and to investigate differences in meal patterns between age groups, sex and socio-demographic factors. Furthermore, we will investigate if differences in meal patterns are associated with estimated energy and nutrient intake in relation to average requirements (AR) of folate, Fe, Se, vitamin D and recommendations for fibre, whole grains, saturated fat and free sugars^(30)^.

## Methods

### Study population

This cross-sectional study consists of a secondary analysis of two different national dietary surveys, Riksmaten Adults 2010–2011^(31)^ and Riksmaten Adolescents 2016–2017^(32)^, both conducted by the Swedish Food Agency. Participants registered their food and drink intake for 3–4 d, including both weekdays and weekends, and completed questionnaires on background characteristics. Some participants also completed blood and urine samples, but biomarkers were not used in this study.

### Riksmaten adults 2010–2011

Riksmaten adults was conducted during 2010 and 2011 in Sweden. A random representative sample based on sex, age and region in the Swedish population was drawn by Statistics Sweden. In total, 5003 people were invited, and 2268 people between ages 18 and 80 years participated, of which 1796 (36 %) had complete dietary information. The participants in the study were more likely to have a higher education and to be born in Sweden than the general Swedish population. Diet was recorded in the validated, web-based food dairy, the Riksmaten method, for four consecutive days^(33,34)^. The majority of the individuals completed all food diary days (98 %), but participants with 2 and 3 valid food diary days were included in the analytical sample. The first day of the food diary was randomised to a weekday (62 %) and Friday, Saturday and Sunday (12 % respectively). The participants could choose from predefined meal types ‘Breakfast’ ‘Lunch’ ‘Dinner/evening meal’ ‘Other eating’ in the web tool and intake could be registered at 15-minute intervals. The food list and food composition database in the web tool were specific to the survey (The Swedish Food Composition Database, version Riksmaten adults 2010–2011). The web tool enables automatic estimation of energy and nutrient intake. The mean intake per day was calculated for each participant.

Participants also completed a questionnaire on demographic and lifestyle factors. Height, weight, education level and smoking status were included as variables in this study.

### Riksmaten adolescents 2016–2017

Riksmaten Adolescents was a school-based dietary survey on children and adolescents. It was carried out in Sweden during 2016 and 2017 in school grades 5 (11–12 years), 8 (14–15 years) and 11 (17–18 years)^(32)^. The ages ranged from 10 to 21 as some participants (*n* 85) were younger and some older (*n* 17) than the normal ages for school years 5 and 11, respectively. Schools were selected by Statistics Sweden based on school grades, type of municipality, public or charter school and geographical spread. 5151 adolescents were invited, and 3477 agreed to participate. The 2967 participants with complete dietary information from 3 days were included in this study. The sample is overall representative of Swedish adolescents^(32)^. The instructions on the validated web tool (RiksmatenFlex) and the first parts of the dietary assessment took place at a school visit by field staff from Swedish Food Agency^(35)^. Participants completed two web-based 24-h recalls and 1 day food diary as well as answered questionnaires, underwent height and weight measurements and tracked and reported physical activity. The first day of the dietary assessment was a 24-hour recall the day before the school visit, followed by a food record on the day of the school visit. The last 24-hour recall day was randomly selected to occur 2–7 d after the first recall day. Both weekdays and weekends were included for a proportional distribution of all days of the week in the sample. The food list and food database in the web tool were specific to the survey (The Swedish Food Composition Database, version Riksmaten Adolescents 2016–2017). When recording their food intake, participants could choose from the predefined meals ‘Breakfast’, ‘Lunch’, ‘Dinner/Evening meal’, ‘Snack’, ‘Other eating’ and ‘Drinks only’. The eating occasions (EO) were tracked at clock times with 1 h slots. During the school visit, the participants who completed blood and urine samples (*n* 1105) were offered fruit and juice.

### Misreporting

To evaluate under- and overreporting, the resting energy expenditure (REE) was calculated with equations using weight and age^(36)^. Reported EI was compared with calculated REE as the ratio EI:REE. Cut-offs for under- and overreporting were calculated as proposed by Black^(37)^ and the physical activity level 1·67 was used for adults and 1·4 for adolescents. The physical activity level values were based on the group averages of the different datasets. The cut-offs were calculated to 0·93 as the lower limit and 3·01 as the highest for adults and 0·83 and 2·35 for adolescents. Participants without weight data could not be included in this evaluation (*n* 32). 367 (12 %) of the adolescents and 284 (16 %) adults had EI outside the cut-offs and were considered misreporters but were kept in the analytical samples and used for sensitivity analyses (see below).

### Meal pattern variables

Using the data collected in the two dietary surveys, the following variables on meal patterns were defined.

#### Eating occasions

EO were defined as any occasion where food and drink were consumed according the participant-identified classification by Leech *et al.* ^(29)^ A minimum energy content of 50 kcal of an EO was set to match previous research. Another variable – EO_food_ – that excluded all EO with only drinks (e.g. soda, coffee, wine) regardless of energy content, was also defined but was only used for descriptive purposes in Table 2.

#### Frequency of eating

The number of EO/24 h was calculated for each participant and further divided into a three-level categorical variable: 3 or fewer, 4–5 and 6 EO or more. This categorical variable was generated as previously done^(38)^ and the middle group also reflected the mean eating frequency in the present study.

#### Time interval between first and last eating occasion

An average time for the first and last EO was calculated for each participant, and weekdays and weekends were analysed separately. Friday morning was defined as a weekday, while Friday evening was considered a weekend. Sunday morning was a weekend, but Sunday evening was considered a weekday.

#### Earlier:later energy intake

Participants defined as ‘earlier type’ have a greater amount of reported energy between 06.00 and 14.59, and ‘later type’ has a greater amount of energy consumed between 15.00 and 23.59^(12,39)^. A daily ratio for early/late EI was calculated for each participant, with a ratio < 1 indicating a late type and > 1 an early type. A mean ratio per individual was calculated and used. Participants with 0 kcal consumed in one timeslot had kcal imputed to 1 kcal to be able to calculate a ratio.

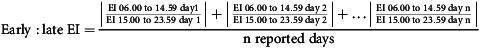




#### Daily eating window

The eating window is the calculated time interval between the first and the last recorded EO across the 24-hour daily cycle. Each participant’s eating window was calculated per day, and then, the mean length over all the registration days was calculated and used in the analysis.

#### Largest meal type

The largest meal type is the meal contributing to the highest proportion of energy during the day. For each participant, the energy percent of each meal type was calculated per day, and a mean for each meal type was then calculated. Four participants were excluded because it was impossible to establish their largest meal type due to identical EI at different meals on the same day, or because they only consumed one meal a day, with different meal types each day.

#### Regularity and meal skipping

Regularity was defined by how similar the number of eating occasions was over the reported days for each individual. The meal frequency for each day and the mean frequency were calculated. We then calculated the absolute difference between the mean and the frequency for each day. This was divided by the reported number of days (3 or 4 d). A higher score indicates a more irregular eating frequency pattern. This method has been used in a previous study^(40)^.

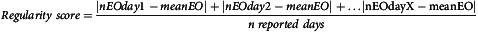




Breakfast consumption was defined according to the registration of the meal type on one or multiple days, regardless of the time the breakfast was recorded. Participants who never ate breakfast or skipped breakfast some days were identified, and a three-level categorical variable on breakfast skipping was generated. ‘Breakfast eaters’ consumed breakfast on all reported days, ‘irregular breakfast skippers’ skipped breakfast on some of the reported days and ‘breakfast skippers’ skipped breakfast on all reported days.

#### Questionnaires and other data

Height and weight were self-reported for adults and measured by staff from Swedish Food Agency for adolescents. BMI (kg/m^2^) was calculated based on height and weight and adult participants were classified according to their BMI status as underweight (BMI < 18·5 kg/m^2^), normal (BMI 18·5–24·9 kg/m^2^), overweight (BMI 25·0–29·9 kg/m^2^) and obese (BMI > 30·0 kg/m^2^). BMI cut-offs for adolescents were based on ISO-BMI from the international obesity task force^(41)^. Educational level was reported by the participants or their parents and defined as the participant’s education level or the highest education of the parents for adolescents. Smoking status was self-reported but not collected for the youngest adolescents 10–12 years. Smoking status was classified as never, daily, former, rarely and I don’t want to answer.

Information on age, sex and living area was collected from the sample drawn by Statistics Sweden for the adult survey and from class lists for the adolescents. Adolescents were grouped into three groups according to school grade. Adults were grouped into the same four age groups as the groups used for the stratified population draw^(31)^. Living area was grouped into urban or rural areas based on the population density of the residential municipality for the adults or school municipality for the adolescents^(31,32)^.

### Statistical methods

All statistical analyses were completed in STATA, version18 (StataCorp). To minimise the risk of false positive results, the significance level was set to 0·01.

Variables were checked for normality using Shapiro–Francia *W*′ test for normality. To evaluate differences in meal patterns between groups within the population, the background variables such as sex, school grade, age groups, living area, education level and smoking status were used. Differences in meal patterns; meal frequency, daily eating window, early:late energy distribution, largest meal type and breakfast skipping were analysed with *t* tests or ANOVA for numerical variables and χ^2^ for categorical variables. Differences between groups in the regularity of eating frequency, the time of the first and last eating occasion and nutrient intakes were tested with Wilcoxon rank-sum test and Kruskal–Wallis test because of non-normality.

### Energy and nutrient intakes

Mean intakes of energy, dietary fibre, whole grains, vitamin D, folate, Fe, Se, saturated fat and free sugars were calculated for each participant and compared between meal patterns. These nutrients were selected since intakes of these nutrients have been shown to be low or too high in the Swedish population^(31,42)^. The absolute intakes were compared with the age-group-specific AR (vitamin D, folate, Fe and Se) or recommendations (fibre, whole grains, saturated fat and free sugars), from the Nordic nutrition recommendations of 2023^(30)^ (online Supplementary Table 4). This was calculated as the percentage of the AR or recommendation and compared between meal frequency categories by completing CI plots displaying means and the 95 % CI in each group. Together with the absolute intakes, all nutrient intakes were also adjusted for EI by calculating intakes per 10 MJ for each participant, presented in the online Supplementary material.

The correlation between meal frequency, eating window and EI was tested with Pearson’s partial correlation with adjustment for sex and age. Energy and nutrient intakes were also compared in each level of breakfast skipping, and for participants that were defined as ‘early-’ and ‘late types’, according to their energy distribution. The differences by groups were investigated with the Kruskal–Wallis test and Wilcoxon rank-sum test.

### Sensitivity analysis

The impact of misreporting was tested by excluding participants with EI outside the previously described cut-offs (0·83–2·35 for adolescents and 0·93–3·01 for adults) in the analyses of the meal pattern variables. Furthermore, a sensitivity analysis was done to evaluate whether participants who completed blood samples were offered fruit and juice (*n* 1105) and had a different meal frequency than the rest of the sample.

## Results

Table 1 presents the background characteristics of the study populations based on school year or age group. A majority (56 %) were girls/women in both samples. The proportion of misreporters was 12 % in adolescents and 16 % in adults.

**Table 1.** Background characteristics of study participants divided by school year and age group, in the Riksmaten adolescents 2016–2017 and Riksmaten adults 2010–2011 surveys

	Riksmaten adolescents 2016–2017 (*n* 2967)*						Riksmaten adults 2010–2011 (*n* 1769)							
	School year						Age group							
	5		8		11		18–30 years		31–44 years		45–64 years		65–80 years	
	*n*	% by survey	*n*	% by survey	*n*	% by survey	*n*	% by survey	*n*	% by survey	*n*	% by survey	*n*	% by survey
*n* (% by survey)	989	33·3	1012	34·1	966	32·6	334	18·6	430	23·9	665	37·0	367	20·4
	Mean	sd	Mean	sd	Mean	sd	Mean	sd	Mean	sd	Mean	sd	Mean	sd
Age	11·5	0·4	14·5	0·4	17·7	0·6	23·7	3·6	37·9	4·0	54·5	5·8	70·4	4·2
	*n*	% by school year	*n*	% by school year	*n*	% by school year	*n*	% by age group	*n*	% by age group	*n*	% by age group	*n*	% by age group
Sex														
Boys/men	455	46	455	45	401	42	131	39	183	43	308	46	169	46
Girls/women	534	54	557	55	565	59	203	61	247	57	357	54	198	54
Misreporting^†^														
Under	69	7	100	10	92	10	74	22	48	11	106	16	45	12
Over	38	4	42	4	26	3	2	1	5	1	2	0	2	1
BMI status^‡^														
Under weight^a^	80	8	74	7	54	6	9	3	7	2	3	0	3	1
Normal weight^b^	676	70	764	76	679	71	214	64	226	53	269	41	156	43
Over weight^c^	182	19	141	14	173	18	55	17	115	27	255	38	147	40
Obesity^d^	35	4	30	3	54	6	55	17	80	19	138	21	61	17
Highest education/parent’s highest education														
Primary	41	4	48	5	35	4	17	5	15	4	91	14	136	37
High school	303	31	288	29	351	36	159	48	160	37	272	41	100	27
University	609	62	598	59	515	53	126	38	222	52	259	39	110	30
None	0		0		0		0		1	0	0		2	1
Missing	36	4	78	8	65	7	32	10	32	7	43	7	18	5
Smoking														
Never	N/A		941	93	669	70	199	66	266	67	286	46	155	44
Daily	N/A		0	0	50	5	26	9	26	7	66	11	26	7
Former	N/A		14	1	50	5	20	7	75	19	251	40	157	45
Rarely	N/A		29	3	168	18	56	19	32	8	20	3	15	4
I don’t want to answer	N/A		25	3	23	2	N/A		N/A		N/A		N/A	
Living area^§^														
Rural	285	29	261	26	404	42	71	21	110	26	205	31	100	27
Urban	704	71	751	74	562	58	236	79	320	74	460	69	267	73

Smoking: Question not asked for school grade 5. Option ‘I don’t want to answer’ is not included in the survey for adults.

* 1: Riksmaten Adolescents 2016–2017, Swedish Food Agency. 2: Riksmaten Adults 2010–2011, Swedish Food Agency.

† Based on cut-offs for EI:REE Adults: 0·93 lower limit, 3·01 upper limit. Adolescents: 0·83 lower limit, 2·35 upper limit.

‡ > 18 years: ^a^BMI < 18·5, ^b^BMI 18·5–24·9 ^c^BMI 25·0–29·9 ^d^BMI > 30·0. < 18 years: ISO-BMI cut-offs, international obesity task force.

§ Urban: Metropolitan, larger cities and surrounding suburbs. Rural: densely built, medium built and sparsely built areas or everything outside metropolitan or larger cities and their suburb surroundings.

### Meal patterns

**Figure 1.**
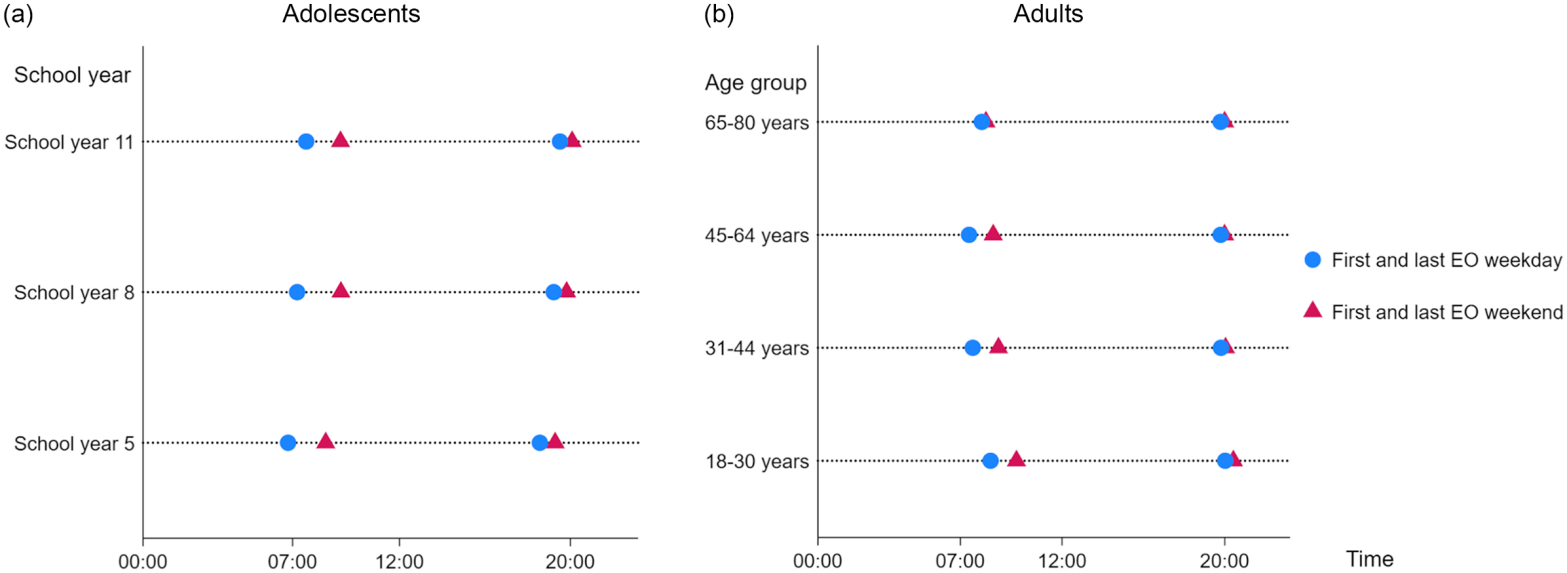
Self-reported first and last eating occasion for (a) adolescents and (b) adults by school year and age group and by weekend and weekday.

Meal pattern variables by survey, sex and school year/age group are presented in Table 2. The eating frequency (number of EO during 24 h) ranged from 1 to 9 in adolescents and 1–10 in adults. The mean number of EO per day was 4·2 (sd 0·9) in adolescents and 4·6 (sd 1·1) in adults. When drinks-only occasions were excluded, the mean number of EO_food_ was 4·1 (sd 0·9) in adolescents and 4·5 (sd 1·1) in adults. The eating window was for adolescents on average distributed between 07.12 and 19.06 on weekdays while on weekends between 09.00 and 19.44 (Figure 1(a)). For adults, it took place between 07.47 and 19.50 on weekdays and 08.49 and 20.04 on weekends (Figure 1(b)). This resulted in an average eating window per 24 h for adolescents of 11.9 h (sd 1·7) and 12.0 h (sd 1·9) for adults. Only 2 % of all EO took place between 23.00 and 05.00 in both adolescents and adults. Around two-thirds of participants in both surveys (adolescents 63 %, adults 65 %) had a greater proportion of energy earlier in the day, that is before 15.00, than later. Only a few adults, 1 % (*n* 15) did not report eating breakfast on any day, while 9 % (*n* 164) skipped breakfast at least one day. In adolescents, it was more common to skip breakfast where 3 % (*n* 77) did not report breakfast on any day and 17 % (*n* 514) skipped breakfast on at least one day.

**Table 2.** Meal pattern variables divided by school year, age group and sex, in the Riksmaten adolescents 2016–2017 and Riksmaten adults 2010–2011 surveys

		Riksmaten adolescents 2016–2017											Riksmaten adults 2010–2011											
		School year						Sex					Age group								Sex			
		5		8		11		Girls		Boys			18–30 years		31–44 years		45–64 years		65–80 years		Women		Men	
*n*		989		1012		966		1656		1311			334		430		665		367		1005		791	
		Mean	sd	Mean	sd	Mean	sd	Mean	sd	Mean	sd		Mean	sd	Mean	sd	Mean	sd	Mean	sd	Mean	sd	Mean	sd
Number of EO, drinks only excluded	^*^	4·1	0·8	4·2	0·9	4·1	1·0	4·2	0·9	4·0	0·9	^* †^	4·1	1·1	4·5	1·0	4·6	1·0	4·7	1·0	4·6	1·0	4·3	1·0
Number of EO, drinks only included	^*^	4·2	0·8	4·3	0·9	4·3	1·0	4·3	0·9	4·1	0·9	^* †^	4·3	1·1	4·6	1·1	4·7	1·1	4·8	1·0	4·7	1·0	4·5	1·2
Eating window hours	^†^	11·7	1·3	11·9	1·7	11·9	2·0	11·8	1·6	11·9	1·8	^†^	11·6	2·3	12·1	1·7	12·3	1·8	11·9	1·7	11·9	1·8	12·1	2·0
Regularity score^§^	^* †^	0·6	0·4	0·6	0·4	0·6	0·4	0·6	0·4	0·6	0·4	^†^	0·6	0·3	0·6	0·3	0·6	0·4	0·5	0·4	0·6	0·3	0·6	0·4
		*n*	% by school year	*n*	% by school year	*n*	% by school year	*n*	% by sex	*n*	% by sex		*n*	% by Age group	*n*	% by Age group	*n*	% by Age group	*n*	% by Age group	*n*	% by sex	*n*	% by sex
Early:late EI^‡^	^†^											^†^												
Early type		705	71	612	61	548	57	1028	62	837	64		193	58	288	67	439	66	254	69	667	67	507	64
Late type		284	29	400	40	418	43	628	38	474	36		141	42	142	33	226	34	113	31	338	34	284	36
Largest meal type	^* †^											^†^												
Breakfast		73	7	58	6	45	5	192	6	74	6		25	8	27	6	40	6	38	10	73	7	57	7
Lunch		244	25	216	21	270	28	396	24	334	26		97	29	131	31	151	23	124	34	279	28	224	28
Dinner/Evening meal		511	52	489	48	463	48	777	47	686	52		208	62	271	63	468	70	204	56	647	64	504	64
Snack/Other eating		56	6	100	10	55	6	144	9	67	5		0	0	0	0	0	0	0	0	0	0	0	0
Drinks only		105	11	149	15	123	13	232	14	145	11		3	1	0	0	6	1	1	0	5	1	5	1
Breakfast skipping (at least on one day)	^†^	90	9	211	21	290	30	327	20	264	20	^†^	81	24	37	9	48	7	13	4	96	10	83	11

* Significant difference between sexes (*P* value < 0·01).

† Significant difference between school years or age groups (*P* value < 0·01).

‡ Distribution of energy intake during the day. Early types have most of their energy intake consumed between 06.00–14.59 and late types between 15.00–23.59.

§ Regularity of eating frequency. A higher regularity score equals to a more irregular eating frequency pattern.

#### Differences in meal patterns by sex

Meal patterns differed by sex in both adolescents and adults (Table 2). Women/girls had a higher eating frequency than men/boys (*P* < 0·001 both comparisons). Adult women also tended to start eating slightly later in the day than men (07.40 *v*. 07.50; *P* = 0·03). It was also more common for adolescent girls to have a snack/other eating/drinks only, as their largest meal type compared with boys (23 % *v*. 16 %; *P* < 0·001).

#### Differences in meal patterns by school year or age group

The number of eating occasions differed significantly by age for adults (Table 2), where the number of EO was higher in older age groups. The time of first and last eating occasion of the day also differed in both adolescents (Figure 1(a)) and adults (Figure 1(b)). Breakfast skipping and being a late type eater were more common in school years 8 and 11, and for adults in the age group 18–30 years, compared with other age groups (Table 2).

#### Differences in meal patterns by other background characteristics

For adolescents, having at least one parent with a university education was associated with an earlier first meal (07.06) and a later last meal (19.09) on weekdays, compared with adolescents in families where the highest education level was primary school (07.36 and 18.41; *P* = 0·002). This means that the eating window was also on average about 1 hour longer (11.1 h *v*. 12.0 h; *P* < 0·001). These adolescents also had a higher eating frequency, 4·2 EO compared with 3·7 EO (*P* < 0·001). Breakfast skipping was more common in households with primary school education, 34 % (*n* 42) as well as high school education 23 % (*n* 212) compared with university education 17 % (*n* 284) (*P* < 0·001). No differences in meal patterns by education level were observed in adults.

Adults living in larger cities had a later distribution of their eating window on weekdays, with first and last meals later than people living in more rural areas. Similarly, adults living in larger cities were more often late types (37 % *n* 487) than people living in more rural areas (28 % *n* 135) (*P* < 0·001). No differences in meal patterns by living area were observed in adolescents.

People who reported smoking daily reported a lower eating frequency, 4·1 EO compared with non-smokers 4·6 EO (*P* < 0·001). The eating window was also shorter for smokers compared with non-smokers (11.5 and 12.2 h, respectively; *P* < 0·001). Breakfast skipping was also more common for adults who smoked daily, 22 % (*n* 32) compared with non-smokers 8 % (*n* 125). A similar pattern regarding breakfast skipping was observed for adolescents who smoked daily (*n* 29) where 58 % were breakfast skippers, compared to 21 % (*n* 350) among those who have never smoked (*P* < 0·001).

### Energy and nutrient intake

**Figure 2.**
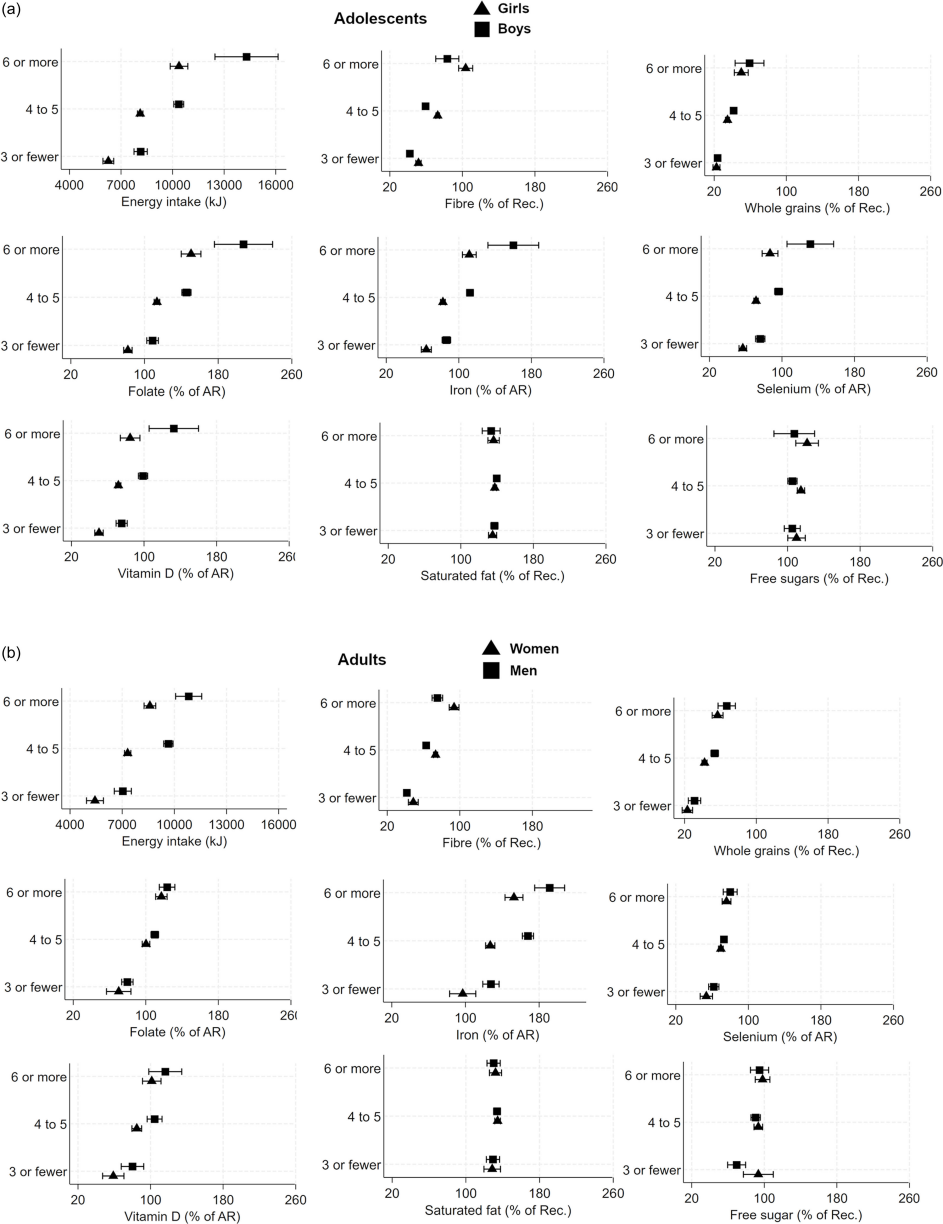
Mean and 95 % CI for energy intake and percentage of average requirement (AR) or recommended intake (Rec.) of nutrients by eating frequency group and sex, for (a) adolescents and (b) adults. AR and Rec. Rec. intake of saturated fat and free sugar is expressed as max value.

There was a moderate positive correlation between the number of eating occasions and EI with adjustment for age and sex, in both adults (*r* = 0·51 *P* < 0·001) and adolescents (*r* = 0·47 *P* < 0·001). Eating window also correlated with EI (adults *r* = 0·38 *P* < 0·001; adolescents *r* = 0·33 *P* < 0·001). The intake of macronutrients differed only slightly between the three groups of eating frequency (online Supplementary Table 1). Absolute intakes of nutrients were higher by eating frequency for all investigated nutrients in all age groups (online Supplementary Table 2). To which extent (%), the participants’ estimated nutrient intakes met the AR or recommended intake by each level of eating frequency are presented in Figure 2(a) for adolescents and Figure 2(b) for adults. Results were stratified by sex since women had a higher eating frequency than men. The results revealed differences in the ability to meet the AR or Rec. for various nutrients between the meal frequency groups. Consuming fewer meals made it more challenging to achieve AR and recommendations compared to having a higher eating frequency.

Both adolescents and adults defined as ‘late types’ with higher EI later in the day had higher intakes of free sugars and saturated fat compared to ‘early types’. Late types had overall higher EI than early types in both adolescents and adults (*P* < 0·001).

There were differences in absolute intakes of nutrients between breakfast eaters and breakfast skippers (online Supplementary Table 3). Breakfast skipping was also associated with lower EI and a lower eating frequency. Even though breakfast skippers had lower EI, when energy was controlled for (per 10 MJ), there were still differences in the nutrient intakes. The composition of macronutrients did not differ between groups (online Supplementary Table 1).

#### Sensitivity analysis

The results from the sensitivity analysis regarding misreporting showed smaller differences in meal patterns when misreporters were excluded. The number of eating occasions for adults was 4·8 EO when misreporters were excluded, compared with 4·6 EO for the whole group of adults. The meal frequency differed for adults between valid reporters (4·8 EO) compared with under-reporters (3·7 EO *P* < 0·001) but not to over-reporters (4·6 EO *P* = 0·78) For adolescents, meal frequency was 4·3 EO when misreporters were excluded, compared with 4·2 EO for the whole sample (*P* < 0·001). There was a difference between valid reporters (4·3 EO) and both under-reporters (3·3 EO *P* < 0·001) and over-reporters (4·8 EO *P* < 0·001). The adolescents (*n* 1105) who completed blood and urine samples and were offered fruit and juice had a significantly higher eating frequency with an average of 0·2 additional reported EO (*P* < 0·001). Only minor differences were observed in meal pattern variables when these participants were excluded.

## Discussion

To our knowledge, this is the first time temporal meal patterns of the Swedish population have been investigated to this extent. The results show that the overall meal pattern was four to five meals per day with an average eating window of about 12 h differing between ages, sexes, education levels, smoking status and living area. A higher meal frequency was associated with higher reported EI, suggesting that individuals consuming fewer meals do not compensate with larger portions and vice versa. A lower eating frequency was also associated with lower absolute intakes of several key nutrients such as whole grains, vitamin D and folate, but lower intakes of free sugars and saturated fat. This suggests that eating few meals per day makes it more challenging to meet nutrient requirements than eating more meals per day.

Various research has examined eating frequency but methodology and definitions of eating occasions and eating frequency vary^(16)^. There is also limited research on larger populations as well as in the Swedish context. A smaller Swedish study reported 5·2 EO per day for reference women and 6·1 EO for women with obesity when using a meal pattern questionnaire^(43)^. Another Swedish study reported similar to our findings, a significantly higher eating frequency for women (5·0 EO) than for men (4·8 EO), and most participants reported 4–5 meals per day also using a questionnaire^(38)^. A European study where Sweden was included found about six EO per day with no differences between sexes, but they used a single 24 h recall^(2)^. Studies from other populations like Australia, the UK and the USA have shown similar average eating frequencies as in our study^(12,16)^. However, comparing meal patterns across countries can be challenging due to cultural differences. The fact that different methods are used to define meal patterns might also affect the results.

Skipping breakfast was associated with a lower intake of some micronutrients but a higher intake of free sugars, indicating a poorer diet quality. A higher eating frequency was associated with a higher EI, while skipping breakfast was linked to a lower EI. These results are in line with results in a meta-analysis of randomised controlled trials where breakfast skipping was associated with a lower EI and lower body weight^(44)^. Our study has not examined metabolic health-related outcomes from meal patterns, but we see that skipping breakfast and three or fewer meals per day makes it difficult to reach nutrient requirements. Further studies are needed to evaluate the actual health outcomes.

The present analyses showed that adolescents skipped breakfast more often than adults. Younger people between 13 and 30 years also tended to have a later energy peak, with higher EI later in the day, compared with those aged 30 years and older, and younger than 13 years. One explanation for this could be their chronotype, where a late chronotype is more common for adolescents^(45)^. Two recent scoping reviews found that later chronotypes skipped meals more frequently, had later meal times and had a later energy distribution^(46,47)^. Previous research from a cohort of about 100 000 adults in France suggested that a later first- and last meal of the day, that is later than 09.00 and 21.00, was associated with a higher cardiovascular disease risk^(11)^. According to previous research, later eating patterns and skipping meals, which are more common in adolescents, could be health risks in the longer perspective.

The current study found a positive association between meal frequency and intake of energy, fibre, whole grains, folate and free sugars in both samples. This is in line with some previous research^(12,16,43)^. Yet, we have not analysed how specific meal types are correlated with nutrient intake. For example, in Sweden, intake of whole grains might be more associated with breakfast than other meal types because of the foods typically consumed at breakfast. This study does not differentiate between main meal frequency and snack frequency. Snack frequency is sometimes studied in comparison to meal frequency; for example, a study by Leech et al. found a positive association between meal frequency and micronutrient intake but not with snack frequency in Australian adults^(16)^. However, a snack can be both healthy and unhealthy, and what a typical snack is differs between countries and this is important to have in mind when studying snacks, snack foods and making comparisons between countries^(48)^.

There is limited previous research on differences in meal patterns within a population and how these patterns vary based on characteristics like age, sex, smoking status, education and living area. Some studies have investigated specific subgroups, like one German cross-sectional study that examined parental characteristics’ effect on children and adolescents’ meal patterns and found associations between meal frequency and parental employment^(49)^. Children ate fewer meals if both parents worked full time. Another German study found that older adults’ meal patterns differed based on their socio-demographic status. Specifically, irregular eaters were more often single and lived alone^(50)^. Our findings suggest differences in the timing of the first meal of the day according to educational level or parental education. This result should be interpreted with caution as different aspects across the lifespan may influence when the first meal is consumed, for example, school hours, type of job, not working or having retired. Worth noting in the current study is that adolescents who had parents with a lower education level were more likely to skip breakfast. However, exploring these complex interactions further is beyond the scope of this article. Altogether, our results show differences in meal patterns between age, sex, living area, education level and smoking status. The differences within the population provide valuable insights for future research, and when evaluating health outcomes, or setting and targeting public health recommendations for nutritious eating.

The strengths of this study are the extensive and nationally representative sample taken from the Swedish population, spanning between the ages 10–80 years. This breadth allows for the examination of age-related differences across the population. The data on adolescents have been stated to be generalisable to the population^(32)^. Also for the adults, the sampling by Statistics Sweden aimed to be representative of the population but the participating subjects were more often higher educated and there was a low representation of people born abroad^(31)^. Moreover, the dietary assessment was done with validated web-based methods, which is a strength. The dietary registrations are used to give detailed information including meal types, clock times and actual food intake and offer a valuable means of assessing meal patterns compared to studies using questionnaires or food frequency questionnaires.

Some limitations of this study need to be addressed. This study used two different surveys that differ slightly in methods. The diet of adolescents was examined with two 24-hour recall days and one food diary, whereas 4-day food diaries were used for the adults. Another difference was that the exactness of the clock time between eating occasions was 15 min for adults while it was 1 hour for adolescents. Further, long-term dietary habits may not be accurately captured in 3 or 4 days. The adult data used in this study is 15 years old; however, this is the latest available national data on adults in Sweden. Most participants in the present study self-administered their diet assessment, which minimises external coding errors but could be considered a weakness. However, errors introduced by individual participants are random and will be alleviated by the big study size. The evaluation of meal frequency was based on an average without distinguishing between weekdays and weekends. The sensitivity analysis that was conducted in participants who completed blood and urine samples offered fruit and juice showed a significantly higher eating frequency. This means that the actual eating frequency for those adolescents probably is lower on average and that the difference compared to older age groups is even larger. However, as the number of eating occasions increased with age, younger people might be less accurate in reporting all their meals. A sensitivity analysis was conducted regarding misreporting, and no large discrepancies in results were discovered when excluding misreporters from the analyses.

Definitions are often stated as a problematic factor in chrononutritional research. In this study, a participant-identified definition of EO was used, where participants reported the type of eating occasion, which resulted in breakfast times between 02.00 and 18.00. This could affect the results when analysing breakfast or other mealtimes where the meal type is used instead of a clock time. This is a type of classification bias since one participant might report that breakfast was not eaten on a certain day if it’s consumed at 18.00 whereas another person always reports the first meal of the day as breakfast regardless of time. Some previous research has used meal type and a time interval for a better definition of a meal that might be beneficial, but this could also be a limiting factor^(11)^. Chronotype is a variable that is often evaluated in chrononutrition, but we did not have any such data to include which is a weakness. The cross-sectional design of the study limits any conclusions regarding cause and effect. However, for investigating dietary and meal patterns in a population, the cross-sectional design is well suited.

In conclusion, the present study suggests that a higher eating frequency and eating breakfast are associated with a higher intake of whole grains, vitamin D and folate and also makes it more likely to reach nutrient requirements. However, a higher eating frequency was also associated with a higher intake of free sugars. Thus, a low eating frequency or skipping breakfast appears to make it difficult to reach nutrient requirements since people who consumed fewer meals per day did not compensate with larger meals or more nutrient-dense foods. Another observation was that a high eating frequency was associated with a higher EI since skipping breakfast and an early energy peak was associated with lower EI. Meal patterns differ by age, sex, education, smoking status and living area. According to our study and previous research, younger people seem to have less healthy meal patterns. As this is an important population group and we have not examined metabolic health-related outcomes, this needs further investigation. Information about the meal patterns of a population is important knowledge when working with public health since these are factors that affect food, energy and nutrient intakes and can help set guidelines or give valuable insights when working with specific groups in the population.

## Supporting information

Schultz et al. supplementary material Schultz et al. supplementary material

## Supplementary material

For supplementary material/s referred to in this article, please visit https://doi.org/10.1017/S0007114525103462
