# Supplementary material for: Temporal meal patterns in the Swedish population and associations with socio-demographic variables and nutrient intakes: a cross-sectional study
Source: Br J Nutr. 2025 May 26;133(10):1363–73. doi: 10.1017/S0007114525103462 (PMC12237699; doi:10.1017/S0007114525103462)
Supplement: Schultz et al. supplementary material [file S0007114525103462sup001.docx]

## Supplementary material

Supplemental Table 1. Macronutrient intakes by tertiles of eating frequency and breakfast skipping

| **Adolescents** | | | | | | |
| --- | --- | --- | --- | --- | --- | --- |
|  | **Meal frequency** | | | **Breakfast skipping** | |  |
|  | **3 or fewer** | **4 to 5** | **6 or more** | **Breakfast eaters** | **Breakfast skippers** |  |
| Energy% Carbohydrates | 45 | 46 | 48 | 46 | 46 |  |
| Energy% Fat | 35 | 35 | 38 | 35 | 36 |  |
| Energy% Protein | 18 | 16 | 16 | 17 | 16 |  |
| Energy% Saturated fat | 14 | 15 | 14 | 14 | 14 |  |
| Energy% monounsaturated fat | 14 | 14 | 13 | 13 | 14 |  |
| Energy% polyunsaturated fat | 5 | 5 | 5 | 5 | 5 |  |
| **Adults** | | | | | | |
|  | **3 or fewer** | **4 to 5** | **6 or more** | **Breakfast eaters** | **Breakfast skippers** |  |
| Energy% Carbohydrates | 41 | 43 | 45 | 43 | 43 |  |
| Energy% Fat | 34 | 35 | 34 | 35 | 35 |  |
| Energy% Protein | 19 | 17 | 16 | 17 | 17 |  |
| Energy% Saturated fat | 13 | 13 | 13 | 13 | 14 |  |
| Energy% monounsaturated fat | 13 | 13 | 13 | 13 | 14 |  |
| Energy% polyunsaturated fat | 6 | 6 | 6 | 6 | 6 |  |
|  |  |  |  |  |  |  |

|  |  |  |
| --- | --- | --- |

Supplemental Table 2. Energy intake and absolute- and energy adjusted intakes (per 10MJ) of micronutrients by tertiles of Eating frequency

| Adolescents | | | | | | | |
| --- | --- | --- | --- | --- | --- | --- | --- |
|  | **3 or fewer** | | **4 to 5** | | **6 or more** | |  |
| mean EO per 24h | 3.2 | | 4.5 | | 6.1 | |  |
| n | 725 | | 2067 | | 175 | |  |
|  | ***mean*** | ***SD*** | ***mean*** | ***SD*** | ***mean*** | ***SD*** |  |
| kJ | 7312 | 2844 | 9044 | 2884 | 11496 | 3730 |  |
| Fiber g  g/10MJ | 13.9  19.9 | 5.8  6.8 | 19.3  21.9 | 7.4  7.0 | 26.8  24.0 | 9.7  7.4 |  |
| Whole grains g  g/10MJ | 20.9 30.8 | 25.1  36.2 | 33.8  38.9 | 30.3  35.4 | 47.4  43.0 | 33.1  29.7 |  |
| Vitamin D µg  µg/10MJ | 4.8  6.7 | 3.3  3.9 | 6.2  6.9 | 3.7  3.4 | 7.4  6.4 | 4.5  3.1 |  |
| Folate µg  µg/10MJ | 213.9  301.8 | 93.7  97.3 | 277.9  314.6 | 106.4  102.4 | 374.4  329.5 | 154.3  96.2 |  |
| Iron mg  mg/10MJ | 6.9  9.6 | 3.5  3.3 | 8.7  9.7 | 3.6  2.6 | 11.5  10.1 | 4.6  2.2 |  |
| Selenium µg  µg/10MJ | 37.1  51.6 | 20.9  23.2 | 43.6  48.5 | 24.4  21.8 | 61.5  49.3 | 98.9  37.7 |  |
| Saturated fat g  g/10MJ | 26.9  36.2 | 13.0  8.2 | 33.6  36.7 | 13.8  7.3 | 41.7  35.9 | 17.2  7.2 |  |
| Free sugar g  g/10MJ | 47.4  64.1 | 38.4  40.7 | 61.0  66.2 | 42.1  33.7 | 80.1  70.2 | 49.2  33.8 |  |
| Adults | | | | | | | |
|  | **3 or fewer** | | **4 to 5** | | **6 or more** | |  |
| mean EO per 24h | 2.9 | | 4.5 | | 6.1 | |  |
| n | 237 | | 1226 | | 333 | |  |
|  | ***mean*** | ***SD*** | ***mean*** | ***SD*** | ***mean*** | ***SD*** |  |
| kJ | 6393 | 2251 | 8323 | 2408 | 9404 | 2678 |  |
| Fiber g  g/10MJ | 13.6  22.1 | 5.6  7.8 | 19.9  24.6 | 6.8  7.2 | 24.5  26.8 | 8.2  8.2 |  |
| Whole grains g  g/10MJ | 25.1  40.6 | 25.7  39.0 | 42.4  52.5 | 29.9  35.8 | 54.4  59.5 | 33.6  35.2 |  |
| Vitamin D µg  µg/10MJ | 5.4  8.6 | 4.0  6.3 | 7.0  8.5 | 4.8  5.5 | 8.0  8.6 | 5.0  4.8 |  |
| Folate µg  µg/10MJ | 189.7  309.8 | 98.9  139.9 | 261.1  324.8 | 101.7  127.8 | 298.7  327.4 | 90.3  96.2 |  |
| Iron mg  mg/10MJ | 8.3  13.6 | 3.1  6.6 | 10.4  12.7 | 3.7  3.5 | 11.9  12.9 | 4.2  3.6 |  |
| Selenium µg  µg/10MJ | 38.8  63.3 | 17.9  28.3 | 45.8  56.6 | 19.2  23.1 | 49.3  53.3 | 19.7  17.7 |  |
| Saturated fat g  g/10MJ | 22.3  34.3 | 10.7  8.9 | 30.0  35.6 | 12.4  8.6 | 33.3  34.9 | 15.1  9.3 |  |
| Free sugars g  g/10MJ | 30.5  47.3 | 23.4  32.4 | 47.1  55.1 | 30.0  28.6 | 55.0  57.9 | 31.0  26.7 |  |

Supplemental table 3. Energy and nutrient intakes by levels of breakfast skipping.

| Adolescents | | | | | | |
| --- | --- | --- | --- | --- | --- | --- |
|  | **Breakfast eaters** | | **Breakfast skippers** | | P-value |  |
| Eating frequency | 4.4 EO | | 3.7 EO | | **<0.001** |  |
| n | 2376 | | 591 | |  |  |
|  | *Mean* | *SD* | *Mean* | SD |  |  |
| kJ | 8900 | 3046 | *8223* | *3242* | **<0.001** |  |
| Fiber g  *g/10MJ* | 19  *22* | 8  7 | 16  *20* | 7  8 | **<0.001** |  |
| Whole grains g  *g/10MJ* | 34  39 | 31  *35* | 23  30 | 25  38 | **<0.001** |  |
| Vitamin D µg  *µg/10MJ* | 6.3  *7.1* | 3.8  *3.5* | 4.5  *5.7* | 3.1  *3.4* | **<0.001** |  |
| Folat µg  *µg/10MJ* | 278  *319* | 112  *100* | 227  *286* | 107  102 | **<0.001** |  |
| Iron mg  *mg/10MJ* | 8.6  *9.8* | 3.9  *2.8* | 7.5  *9.3* | 3.2  2.5 | **<0.001** |  |
| Selenium µg  *µg/10MJ* | 44  *50* | 36  *24* | 38  *47* | 21  *23* | **<0.001** |  |
| Saturated fat g  *g/10MJ* | 33  37 | 14  7 | 31  37 | 15  8 | **0.66** |  |
| Free sugars  *g/10MJ* | 58  64 | 40  34 | 63  74 | 50  41 | **<0.001** |  |
| Adults | | | | | | |
|  | **Breakfast eaters** | | **Breakfast skippers** | | P-value |  |
| Eating frequency | 4.7 EO | | 3.7 EO | | **<0.001** |  |
| n | 1617 | | 179 | |  |  |
|  | *Mean* | *SD* | *Mean* | SD |  |  |
| kJ | *8356* | *2551* | *7480* | 2709 | **<0.001** |  |
| Fiber g  *g/10MJ* | 20  *25* | 7  8 | 15  *21* | 7  7 | **<0.001** |  |
| Whole grains g  *g/10MJ* | 44  54 | 31  *37* | 26  34 | 27  31 | **<0.001** |  |
| Vitamin D µg  *µg/10MJ* | 7.1  *8.8* | 4.9  *5.6* | 5.2  *6.9* | 3.9  4.3 | **<0.001** |  |
| Folat µg  *µg/10MJ* | 265  *328* | 103  *126* | 205  *281* | 91  95 | **<0.001** |  |
| Iron mg  *mg/10MJ* | 10.6  *12.9* | 3.8  *4.1* | 8.8  *12.0* | 3.4  3.3 | **<0.001** |  |
| Selenium µg  *µg/10MJ* | 46  *57* | 19  *24* | 39  *54* | 17  18 | **0.19** |  |
| Saturated fat g  *g/10MJ* | 30  35 | 13  9 | 27  36 | 13  9 | **0.24** |  |
| Free sugars  *g/10MJ* | 46  54 | 30  28 | 47  60 | 34  36 | **0.013** |  |

Supplemental Table 4. Average requirements (AR) or recommended intakes (Rec.) for nutrients based on Nordic nutrition recommendation 2023.

| **Nutrient** | **Average requirement (AR) or recommended intake (Rec.)** |
| --- | --- |
| **Fiber Rec.** | Women: 25 grams per day  Men: 35 grams per day |
| **Whole grains Rec.** | 90 grams per day |
| **Vitamin D AR** | 7.5 µg per day |
| **Folate AR** | 7-10 years: 160 µg per day  Girls 11-14 years: 220 µg per day  Girls 15-17 years: 240 µg per day  Girls/Women >18 years: 250 µg per day  Boys 11-14 years: 200 µg per day  Boys/Men >15 years: 250 µg per day |
| **Iron AR** | 7-10 years: 7 mg per day  Girls 11-14 years: 10 mg per day  Girls/women 15-50 years: 9 mg per day  Women >51 years: 6 mg per day  Boys 11-17 years: 9 mg per day  Men >18 years: 7 mg per day |
| **Selenium AR** | 7-10 years: 35 µg per day  Girls 11-14 years: 50 µg per day  Girls 15-17 years: 55 µg per day  Girls/Women >18 years: 60 µg per day  Boys 11-14 years: 50 µg per day  Boys/Men >15 years: 70 µg per day |
| **Saturated fat Rec.** | Max 10E% |
| **Free sugars Rec.** | Max 10E% |
